# Respiratory syncytial virus (RSV) disease – new data needed to guide future policy

**DOI:** 10.7189/jogh.05.020101

**Published:** 2015-12

**Authors:** Harry Campbell, Louis Bont, Harish Nair

**Affiliations:** 1 Centre for Global Health Research, Usher Institute of Population Health Sciences and Informatics, University of Edinburgh, Edinburgh, Scotland, UK; 2 Department of PediatricsUniversity Medical Center Utrecht, Utrecht, Netherlands

## Abstract

RSV is the main cause of childhood lower respiratory infections, globally, an important cause of childhood wheeze and may be responsible for a substantial burden of disease in the very elderly and in adults with chronic medical problems, such as COPD. It is thus responsible for substantial healthcare and social costs. There are currently many companies and academic groups developing and testing candidate vaccines and there is an expectation that these will lead to effective and safe vaccines which will be available to health systems globally in the short – medium term. Despite this, there is an incomplete understanding of RSV disease, especially in adult groups, and large scale data are only available from a few countries and settings leading to low levels of awareness of the importance of this pathogen. We discuss the need for widespread national sentinel systems of RSV surveillance and some means by which this could be achieved. These data will be needed by national policy makers and immunisation advisory groups to guide future priority setting and decision making.

In this issue of the *Journal of Global Health* there is a short series of papers on respiratory syncytial virus infection (RSV). RSV is a major cause of morbidity and mortality worldwide and has been estimated to cause about 34 million episodes of acute lower respiratory infections (ALRI) in young children globally each year, with over 3 million severe enough to cause hospitalisation [1]. These episodes are followed by an increased risk of wheeze in later childhood. In addition, the role of RSV in causing disease in adults with chronic medical problems and in the very elderly is ill–defined but may also represent a substantial burden of disease. Reports from Falsey in the USA and a recent study from UK suggest that RSV may be an important cause of primary care consultations, hospital admissions and deaths from cardiopulmonary causes following RSV infection in these adult risk groups, similar to influenza [2].

In 2015, the World Health Organization (WHO) Product Development for Vaccines Advisory Committee (PDVAC), which scans the horizon for likely important vaccine developments in the coming decade, highlighted RSV as “a pathogen for which there is major vaccine pipeline activity, high technical feasibility, and major disease burden in low and middle income countries (LMICs)”. They summarised the current status of vaccine research as of June 2014 [3], and this status is updated by PATH in their RSV vaccine snapshot graphic [4]. A number of different vaccine candidates are being developed and tested in phase 1–3 trials and there is an expectation, echoed by WHO PDVAC that these may become available to health systems in the short – medium term.

Currently, these developments are gaining momentum ahead of action to gather information to raise awareness and inform policy discussions globally. Although RSV disease accounts for very significant health care and social costs globally, there is a low level of recognition of this among government policy makers. Thus, to support these developments there is a need to assemble epidemiological data on RSV through national and other large scale surveillance systems. This could provide data on:

• burden of RSV disease by age group, by key risk groups and by geographical setting;

• RSV seasonality patterns across the world; and

• RSV viral parameters such as subtypes and genotypes.

They could also provide baseline data on hospitalisations from RSV ALRI against which the impact of a future vaccine could be assessed at national scale.

RSV is not currently a notifiable disease and there are no well–established ongoing surveillance systems in existence. A sophisticated Global Influenza Surveillance and Reporting System (GISRS) functions well with data from over 140 National Influenza Centres globally and with the sharing of over 1 million respiratory samples for influenza detection. The paper by Shi and colleagues in this edition of Journal of Global Health demonstrates that about 90% of ALRIs in children in which RSV is detected can be causally ascribed to RSV. Some of the GISRS sites conduct acute surveillance of Severe Acute Respiratory Infections (SARI) in all age groups and although case definitions and sampling procedures are designed to identify episodes of SARI due to influenza there is the potential to adapt this surveillance system to also identify episodes of ALRI due to RSV. Key issues to address in this adaptation would include the differing seasonal pattern of RSV, the different age pattern of RSV disease (with more emphasis on the first 2 years of life) and the differing clinical presentation of cases (with fever absent in up to 80% of cases in children) [5]. Most severe cases of RSV infection, including RSV–related deaths, occur during the first months of life, during which fever is often absent and apnoea may be the presenting or even only symptom, similar to pertussis. These represent major challenges to overcome if a parallel system for RSV within GISRS is to be developed. However, this approach perhaps represents the only feasible means by which most LMICs will be able to generate substantial amounts of structured and reasonably representative RSV data which can be used to inform decision making on the priority for future RSV vaccine introduction. These systems will under–estimate true RSV disease burden (due to some children with RSV disease not attending for care in LMIC) but establishing such a surveillance system would represent a quantum leap forward in the availability of health care data on RSV and in data on RSV seasonality from most countries globally.

It is not fully clear how the existing GISRS systems can best be adapted for RSV surveillance but this will at least require the development of separate case definitions and RSV–specific standard operating procedures for identification of the target population, data collection, analysis and reporting. This will require specific study and the new scheme will need to be validated before wide adoption. The proposed global web–based FLuMART platform for sharing epidemiological (FLuID) and virological (FluNet) data could serve as a base from which an RSV information and reporting system could be built. A major advantage of this approach would be the existing excellent linkages to national policy makers globally who already receive weekly reports from GISRS. These developments will need to be supported by RSV reference laboratories which can conduct external quality assurance of participating laboratories. It will be essential to ensure that this development does not degrade the quality, completeness or timeliness of data on influenza since the GISRS data represents a vital public health activity eg, in informing annual influenza vaccine composition and in pandemic preparedness. Thus, it would be prudent to start with pilot projects in a few countries in each global region supported by a few designated RSV reference laboratories and this is the approach currently being proposed by WHO [6].

In high–income countries, in addition to the approach noted above, other options could be feasible. These could include bespoke hospital–based surveillance schemes focused on RSV or, as in Canada (in their Serious Outcomes Surveillance (SOS) and Immunization Monitoring Program ACTive (IMPACT) paediatric surveillance network [7]), be part of a broader hospital surveillance scheme targeting a number of infectious diseases such as rotavirus, pertussis and RSV. Setting up such a new surveillance system would be an expensive and technically demanding challenge which will have to be developed in a step wise manner focussing on training, capacity building and provision of technical resources. In Europe, the European Centre for Disease Control (ECDC) held a meeting of an RSV Task Force in 2003 and more recently a consultation on RSV surveillance in November 2015. The 2003 Task Force concluded that RSV surveillance was feasible in Europe based on the experience of reporting RSV disease episodes in 6 European countries and recommended that future real–time data on RSV should be possible to provide through the European Influenza Surveillance System (EISS) reporting infrastructure [8]. The 2015 consultation reviewed the current status of RSV surveillance in Europe and started discussion with a wide range of national public health agencies on how this could develop in Europe in the next few years. These developments in surveillance will need to be accompanied by investment in new cohort studies to better understand RSV epidemiology and burden of disease.

Reductions in mortality from childhood pneumonia have been the largest single contributor to global falls in post–neonatal child mortality over the past decade and have been due to a variety of factors associated with socio–economic development as well the introduction of new vaccine and other interventions. The development of effective new vaccines and other prevention strategies to tackle RSV have the potential to make major contributions to reducing severe disease and deaths from RSV in children globally. These developments may also have an important impact on cardio–respiratory sequelae due to RSV associated with from the growing tide of non communicable diseases among middle aged and elderly adults globally. It is important that structured data gathering systems are put in place now to gather the essential data that will be required to provide a secure evidence base to guide national, regional and global policy decisions in the near future.
